# Comparative Genomics on Cultivated and Uncultivated Freshwater and Marine “*Candidatus* Manganitrophaceae” Species Implies Their Worldwide Reach in Manganese Chemolithoautotrophy

**DOI:** 10.1128/mbio.03421-21

**Published:** 2022-03-14

**Authors:** Hang Yu, Grayson L. Chadwick, Usha F. Lingappa, Jared R. Leadbetter

**Affiliations:** a Division of Geological & Planetary Sciences, California Institute of Technology, Pasadena, California, USA; b Division of Engineering & Applied Science, California Institute of Technology, Pasadena, California, USA; Harvard University

**Keywords:** autotroph, lithotroph, chemolithoautotroph, manganese oxide, manganese carbonate, *Nitrospirae*, *Nitrospirota*, Mn, Mn^2+^, Mn(II)

## Abstract

Chemolithoautotrophic manganese oxidation has long been theorized but only recently demonstrated in a bacterial coculture. The majority member of the coculture, “*Candidatus* Manganitrophus noduliformans,” is a distinct but not yet isolated lineage in the phylum *Nitrospirota* (*Nitrospirae*). Here, we established two additional MnCO_3_-oxidizing cultures using inocula from Santa Barbara (California) and Boetsap (South Africa). Both cultures were dominated by strains of a new species, designated “*Candidatus* Manganitrophus morganii.” The next most abundant members differed in the available cultures, suggesting that while “*Ca.* Manganitrophus” species have not been isolated in pure culture, they may not require a specific syntrophic relationship with another species. Phylogeny of cultivated “*Ca.* Manganitrophus” and related metagenome-assembled genomes revealed a coherent taxonomic family, “*Candidatus* Manganitrophaceae,” from both freshwater and marine environments and distributed globally. Comparative genomic analyses support this family being Mn(II)-oxidizing chemolithoautotrophs. Among the 895 shared genes were a subset of those hypothesized for Mn(II) oxidation (Cyc2 and PCC_1) and oxygen reduction (TO_1 and TO_2) that could facilitate Mn(II) lithotrophy. An unusual, plausibly reverse complex 1 containing 2 additional pumping subunits was also shared by the family, as were genes for the reverse tricarboxylic acid carbon fixation cycle, which could enable Mn(II) autotrophy. All members of the family lacked genes for nitrification found in *Nitrospira* species. The results suggest that “*Ca.* Manganitrophaceae” share a core set of candidate genes for the newly discovered manganese-dependent chemolithoautotrophic lifestyle and likely have a broad, global distribution.

## INTRODUCTION

Members of the bacterial phylum *Nitrospirota* (formerly *Nitrospirae*) are best known for performing difficult physiologies that exploit the utilization of unusually high potential electron donors or low potential electron acceptors (1, 2). Cultivated organisms representing this phylum cluster within 4 clades. Order *Nitrospirales* (formerly genus *Nitrospira*) plays an important role in the nitrogen cycle, carrying out nitrite oxidation (3, 4) and complete ammonium oxidation to nitrate (5, 6). Class *Leptospirilla* (formerly genus *Leptospirillum*) thrive in low-pH environments oxidizing iron (7). Class *Thermodesulfovibria* (formerly genus *Thermodesulfovibrio*) includes high-temperature dissimilatory sulfate reducers (8), some with the capacity of S disproportionation (9), as well as uncultivated magnetotactic bacteria (10). Recently, a bacterial coculture was demonstrated to perform Mn(II) oxidation-dependent chemolithoautotrophic growth (11). This metabolism was attributed to a member of a previously uncultivated clade of *Nitrospirota*, “*Candidatus* Manganitrophus noduliformans” strain Mn1, given that the minority member in the coculture, *Ramlibacter lithotrophicus* (*Comamonadaceae*; formerly within the *Betaproteobacteria*, now within *Gammaproteobacteria*) could be isolated yet would not oxidize Mn(II) alone (11). Based on 16S rRNA gene phylogeny, relatives of strain Mn1 were identified around the world and in diverse freshwater ecosystems (11). However, whether or not these relatives share the same Mn(II) oxidation metabolism was not something that could be gleaned from their rRNA genes.

Mn is the third most abundant redox-active metal in the Earth’s crust and is actively cycled (12–14). Microbial reduction of Mn oxides for growth has been demonstrated in numerous bacterial and archaeal phyla (14–18). The notion that microbial oxidation of Mn(II) with O_2_ could serve as the basis for chemolithoautotrophic growth was first theorized decades ago (13, 14, 19, 20). This metabolism, while energetically favorable (Δ*G*°′ = −68 kJ/mol Mn), poses a biochemical challenge to the cell because of the high average potential of the two Mn(II)-derived electrons [Mn(II)/Mn(IV), E°′ = +466 mV (11)]. These electrons would need their redox potential to be lowered by nearly a full volt in order to reduce the ferredoxin (*E*°′ = −320 to −398 mV [21]) employed in their CO_2_ fixation pathway (11). This is a larger and more significant mismatch in redox potential than similar chemolithotrophic metabolisms, such as nitrite or iron oxidation [NO_2_^−^/NO_3_^−^, E°′ = +433 mV (21); Fe(II)/Fe(III), E°′ of ∼0 mV (22)]. Based on deduced homology with characterized proteins involved with Fe(II) oxidation or aerobic metabolism, genes for 4 putative Mn-oxidizing complexes and 5 terminal oxidases were identified in strain Mn1 and proposed as candidates for energy conservation via electron transport phosphorylation (11). Remarkably, gene clusters for 3 different complex I exist in strain Mn1 and could facilitate the otherwise endergonic coupling of Mn(II) oxidation to CO_2_ reduction, allowing for autotrophic growth via reverse electron transport, i.e., expending motive force to drive down electron reduction potential (11). The apparent redundancy of diverse novel complexes in several members of the family remains puzzling. It seems clear that the identification and analysis of additional strains and genomes of Mn(II)-oxidizing chemolithoautotrophs could shed light on the complexes essential for this newfound mode of metabolism.

The ever-increasing number of metagenome-assembled genomes (MAGs) available in the databases provides for an unprecedented opportunity to learn about the gene content and potential functions of many uncultured microorganisms. However, cultivation remains critical to forming interconnections between the genomes of both cultured and uncultivated microbes and their metabolisms. Here, we successfully established new enrichment cultures performing chemolithoautotrophic Mn oxidation from two disparate environmental inoculum sources. By comparing the MAGs of the most abundant organisms present in these enrichments, members of the *Nitrospirota*, as well as 66 newly and publicly available MAGs in the databases belonging to *Nitrospirota* clades with unexamined metabolisms, we gain insight into a core set of candidate genes for facilitating chemolithoautotrophic Mn oxidation as well as the phylogenetic and geographic distribution of known and putatively Mn-oxidizing *Nitrospirota*.

## RESULTS

### Reproducible cultivation of Mn-oxidizing chemolithoautotrophs.

“*Ca.* Manganitrophus noduliformans” strain Mn1 was accidentally enriched in tap water (11). Using the defined Mn(II) carbonate medium in this previous study (11), new Mn-oxidizing enrichment cultures were successfully established from two distinct sample sources. One inoculum was material from a Mn oxide-containing rock surface near Boetsap, Northern Cape, South Africa (South Africa enrichment), and the other inoculum was material from an iron oxide microbial mat in Santa Barbara, CA, USA (Santa Barbara enrichment). While the new enrichments grew in the same defined freshwater medium, they exhibited different temperature optima. The South Africa enrichments initially grew at 28.5°C, although they oxidized Mn(II) faster at 32°C, similar to the previous enrichment from the Pasadena drinking water distribution system (Pasadena enrichment) (11). The Santa Barbara enrichments grew at 28.5°C but not at 32°C. Otherwise, the three enrichment cultures exhibited similar phenotypes, including the formation of small Mn oxide nodules. These results indicate that the defined Mn(II) carbonate medium can successfully be employed during intentional, directed attempts to cultivate Mn-oxidizing chemolithoautotrophs from diverse terrestrial and aquatic freshwater environments.

### Community analysis of Mn-oxidizing enrichment cultures from three origins.

**FIG 1:**
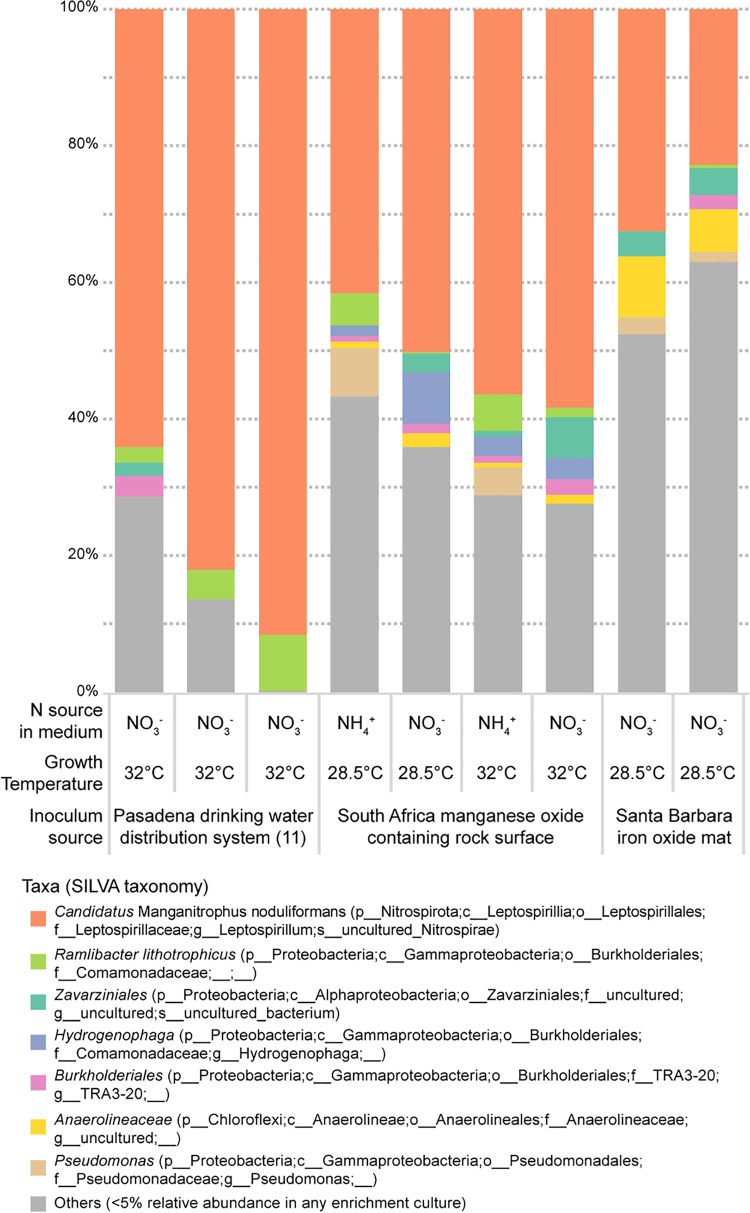
Community analysis of manganese-oxidizing enrichment cultures using partial 16S rRNA gene amplicon sequencing. Taxonomic classification is based on the SILVA small subunit rRNA database v138. Detailed taxon relative abundances can be found in Table S1.

As was the case with cultures of “*Ca.* M. noduliformans,” repeated attempts to identify single colonies of the lithotrophs responsible for Mn oxidation were not successful on an agar-solidified, defined Mn(II) carbonate medium. Sequencing of partial 16S rRNA genes amplified from the liquid cultures revealed differences in community structures between the Mn-oxidizing enrichments. The most abundant microorganism from the South Africa and Santa Barbara enrichments belonged to the same taxon as the previously described “*Ca.* M. noduliformans” (Fig. 1). However, the identities of the next most abundant members of the communities differed. The previously described Pasadena enrichment containing “*Ca.* M. noduliformans” had *Ramlibacter lithotrophicus* as the second most abundant member throughout the enrichment refining process (see Table S1 in the supplemental material). *R. lithotrophicus* could be isolated from the enrichment using the same defined medium but with other electron donors such as succinate and hydrogen but could not oxidize Mn(II) as an isolate (11). Organisms belonging to the same taxon as *R. lithotrophicus* were present in the South Africa enrichments, varying from 2 to 28 in rank abundance, but were not abundant in Santa Barbara enrichments (<0.5% relative abundance) (Fig. 1 and Table S1). In the South Africa enrichments, the second most abundant member varied between a Pseudomonas species (*Gammaproteobacteria*), a member of the *Zavarziniales* (*Alphaproteobacteria*), *R. lithotrophicus*, and *Hydrogenophaga* (a *Comamonadaceae* closely related to *R. lithotrophicus*) (Fig. 1). In the Santa Barbara enrichments, the second most abundant member was a member of the *Anaerolineaceae* (phylum *Chloroflexi* or *Chloroflexota*; Fig. 1). Changing the incubation temperature did not affect the identities of the 3 most abundant taxa in the South Africa enrichments (Fig. 1). However, the choice of nitrogen source in the medium resulted in a shift in community member relative abundances (Fig. 1). Notably, the only other shared organism between South Africa, Santa Barbara, and Pasadena enrichments with >1% relative abundance was a member of the *Zavarziniales* (Fig. 1 and Table S1). Its relative abundance markedly increased when the South Africa enrichments were grown in medium with nitrate instead of ammonia as the nitrogen source. Overall, while the community composition varied between the Mn-oxidizing enrichments, strains of “*Ca.* Manganitrophus” were consistently the most abundant species in all such cultures.

10.1128/mbio.03421-21.4 TABLE S1 Community analysis of Mn-oxidizing enrichment cultures using partial 16S rRNA gene amplicons. Download Table S1, XLSX file, 0.04 MB. Copyright © 2022 Yu et al. 2022 Yu et al. https://creativecommons.org/licenses/by/4.0/ This content is distributed under the terms of the Creative Commons Attribution 4.0 International license.

### Expansion of MAGs of cultivated and environmental Mn-oxidizing *Nitrospirota*.

**FIG 2:**
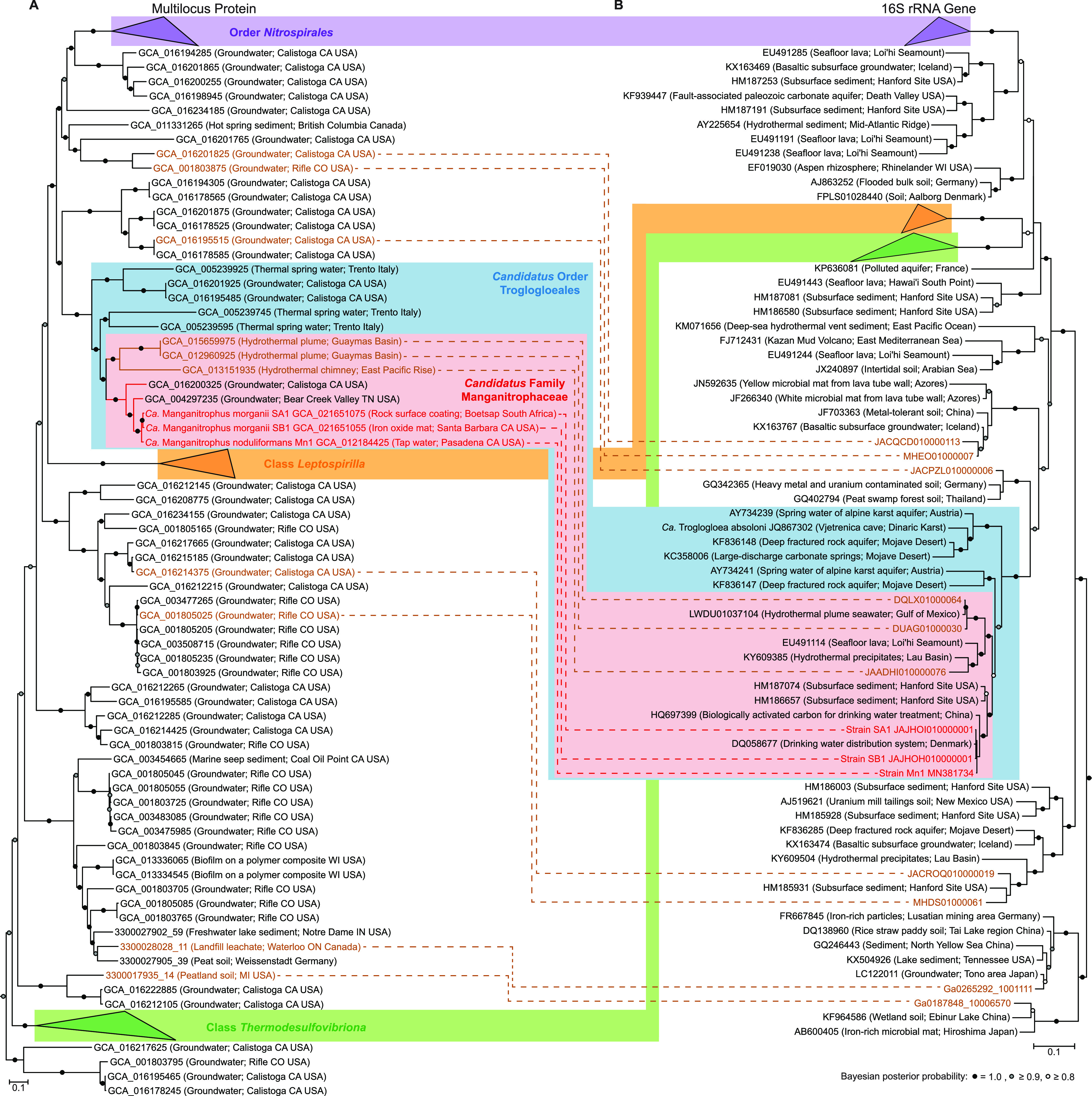
Phylogenetic analysis of the bacterial phylum *Nitrospirota*. (A) Multilocus phylogram, based on a Bayesian analysis of 5,040 aligned amino acid positions concatenated from 120 bacterial protein markers. (B) 16S rRNA gene phylogram, based on a Bayesian analysis of 1,508 aligned nucleotide positions. For both panels A and B, NCBI accession numbers or IMG contig identifiers for the genome assemblies or 16S sequences are in the node names, with their source environments shown in parentheses. Two phylograms can be linked by the genomes assemblies that contain 16S rRNA genes, with environmental metagenomes in brown and manganese-oxidizing enrichment cultures in red. Previously described taxonomic groups based on GTDB taxonomic classifications and the proposed taxonomic groups are grouped by color.

We performed shotgun metagenomic sequencing on two of the new Mn-oxidizing enrichments in order to gain phylogenetic and functional insights into the newly cultivated “*Ca.* Manganitrophus” strains. We reconstructed high-quality metagenome-assembled genomes (MAGs) (>97% completeness, <5% contamination) (23) of the most abundant organism from each metagenome (Table S1). We refer to these MAGs as strains SA1 and SB1 to indicate that they originated from South Africa and Santa Barbara, respectively. Both genome and 16S rRNA gene phylogenies confirmed that strain SA1 and strain SB1 were related to the previously characterized “*Ca.* M. noduliformans” strain Mn1 (Fig. 2). Based on their average nucleotide identities (ANI) and using 95% ANI as a possible metric for species delineation (24–26), strains SA1 and SB1 were provisionally considered to represent distinct strains of the same species (96% ANI). Both could be considered a different species than strain Mn1 (94% ANI) (Table S3). The genome sizes of these 2 new strains were smaller (4.3 Mb) than that of strain Mn1 (5.2 Mb) (Table S2). The arrangements of homologous regions in strains SA1 and SB1 were similar (Fig. S1a) but were different from that of strain Mn1 (Fig. S1b). These differences were also observed at the deduced protein level, with strains SA1 and SB1 more closely related to each other than to strain Mn1 (Table S4). These variations in the proteins were not concentrated in one genomic region but instead scattered throughout the genome (Fig. S1c). Further, *de novo* gene clustering showed that strains SA1 and SB1 shared more genes with each other than with strain Mn1 (Fig. S1d). Altogether, our results support strains SA1 and SB1 as distinct species, which we designate “*Candidatus* Manganitrophus morganii” (Text S1). These 3 cultivated “*Ca.* Manganitrophus” strains in two different species provide a basis to examine the phylogenetic and genomic diversity of their shared metabolism, namely, Mn-oxidizing chemolithoautotrophy.

10.1128/mbio.03421-21.1 TEXT S1 Taxonomic description of “*Candidatus* Manganitrophus morganii,” sp. nov. Download Text S1, DOCX file, 0.01 MB. Copyright © 2022 Yu et al. 2022 Yu et al. https://creativecommons.org/licenses/by/4.0/ This content is distributed under the terms of the Creative Commons Attribution 4.0 International license.

10.1128/mbio.03421-21.2 FIG S1 Comparison of metagenome-assembled genomes of 3 cultivated “*Candidatus* Manganitrophus” strains. (A) Reciprocal mapping of the nucleotide sequences shows similar ordering of homologous regions between strain SA1 and strain SB1. (B) Reciprocal mapping of the nucleotide sequences shows differences in the ordering of homologous regions between strain Mn1 and strains SA1/SB1. (C) Comparing the protein sequences of strain Mn1 to genomes of strains SA1 (inner circle) and SB1 (outer circle) reveals that the nucleotide differences are observed in the amino acids throughout the genomes. (D) Comparison of *de novo* gene clusters reveals that strains SA1 and SB1 share more gene content with each other than with strain Mn1 despite the comparatively close proximity of Santa Barbara and Pasadena. Download FIG S1, JPG file, 0.5 MB. Copyright © 2022 Yu et al. 2022 Yu et al. https://creativecommons.org/licenses/by/4.0/ This content is distributed under the terms of the Creative Commons Attribution 4.0 International license.

10.1128/mbio.03421-21.5 TABLE S2 Assembly and genome statistics of two new “*Ca.* Manganitrophus morganii” strains in enrichment cultures. Download Table S2, DOCX file, 0.01 MB. Copyright © 2022 Yu et al. 2022 Yu et al. https://creativecommons.org/licenses/by/4.0/ This content is distributed under the terms of the Creative Commons Attribution 4.0 International license.

10.1128/mbio.03421-21.6 TABLE S3 Average nucleotide identity (ANI) comparison of genomes and metagenome-assembled genomes in the family “*Candidatus* Manganitrophaceae”. ANI values less than 0.75 are unreliable and therefore given a value of 0 instead. The MAG IDs of the marine genus are in brown. Download Table S3, DOCX file, 0.01 MB. Copyright © 2022 Yu et al. 2022 Yu et al. https://creativecommons.org/licenses/by/4.0/ This content is distributed under the terms of the Creative Commons Attribution 4.0 International license.

10.1128/mbio.03421-21.7 TABLE S4 Average amino acid identity comparison of genomes and metagenome-assembled genomes in the family “*Candidatus* Manganitrophaceae.” The MAG IDs of the marine genus are in brown. Download Table S4, DOCX file, 0.01 MB. Copyright © 2022 Yu et al. 2022 Yu et al. https://creativecommons.org/licenses/by/4.0/ This content is distributed under the terms of the Creative Commons Attribution 4.0 International license.

In addition to reconstructing MAGs from Mn-oxidizing enrichments, we also analyzed publicly available MAGs in the phylum *Nitrospirota*. We screened for MAGs that did not belong in the three characterized clades, namely, *Nitrospirales*, *Leptospirilla*, and *Thermodesulfovibria*. As of 26 March 2019, only 3 MAGs had met this taxonomic criteria with completeness of >50% and contamination of <5% (11). However, as of 30 March 2021, 64 new public high-quality (>90% completeness, <5% contamination) and 2 medium-quality (>50% completeness, <10% contamination) MAGs meeting these taxonomic criteria had become available (Table S5). These 66 MAGs allowed for a much more detailed phylogenomic view into the uncultivated *Nitrospirota* and their potential ability to oxidize Mn.

10.1128/mbio.03421-21.8 TABLE S5 Genome statistics of the publicly available metagenome-assembled genomes used in this study. The MAG IDs for the family “*Candidatus* Manganitrophaceae” are highlighted in pink, with the text of the marine genus in brown; other members in the order “*Candidatus* Troglogloeales” are highlighted in blue. Download Table S5, DOCX file, 0.02 MB. Copyright © 2022 Yu et al. 2022 Yu et al. https://creativecommons.org/licenses/by/4.0/ This content is distributed under the terms of the Creative Commons Attribution 4.0 International license.

### 16S rRNA gene and multilocus protein phylogeny reveal robust taxonomic groups.

The available MAGs provide a phylogenetic resolution that matches the traditionally employed 16S rRNA genes (Fig. 2). The MAGs were spread out across different phylogenetic clusters within the phylum (Fig. 2A). Using the 14 MAGs that also contained 16S rRNA genes, we were able to link the genome phylogeny to the 16S rRNA gene phylogeny and observed similar clusterings between the two phylogenetic approaches (Fig. 2). The 3 cultivated strains all resided within the genus “*Ca.* Manganitrophus.” Other members of “*Ca.* Manganitrophus,” based on either their genomes or 16S rRNA genes, were from terrestrial, aquatic, and engineered environments and all freshwater in origin (Fig. 2). Our phylogeny revealed a sister genus of marine origin (Fig. 2). Together, these two genera form a coherent and well-supported phylogenetic clade, here termed family “*Candidatus* Manganitrophaceae” (Fig. 2).

Previously, the class “*Candidatus* Troglogloea” was proposed to encompass strain Mn1 and “*Candidatus* Troglogloea absoloni” (an uncultivated species from Vjetrenica cave in the Dinaric Karst) based on their 16S rRNA gene phylogeny (11). Based on our new phylogenomic analysis, we propose that the order “*Ca.* Troglogloeales” includes the family “*Ca.* Manganitrophaceae,” “*Ca.* T. absoloni,” and its relatives (Fig. 2), together constituting a sister group distinct from the order *Nitrospirales* (which includes the cultivated nitrite and ammonia-oxidizing *Nitrospirota*). These genera, family, and order proposals are consistent with the latest taxonomic classification in the Genome Taxonomy Database (GTDB) release 06-RS202 April 2021 (27, 28), even though GTDB currently contains fewer genomes. Based on the current GTDB taxonomy, both orders “*Ca.* Troglogloeales” and *Nitrospirales* are placed within the class *Nitrospiria*, but this is incongruent with analyses of their 16S rRNA phylogeny (Fig. 2B). Numerous *Nitrospirota* MAGs fall outside the three known groups of *Nitrospirota* (*Nitrospirales*, *Leptosprillia*, and *Thermodesulfovibriona*) and are overrepresented in subsurface and aquatic environments. However, 16S rRNA gene surveys indicate that members of many of the uncultivated clades exist from marine, soil, and sediment environments but are not represented by genomes (Fig. 2B). Overall, while the taxonomic relationship between orders “*Ca.* Troglogloeales” and *Nitrospirales* and the assignment of classes in *Nitrospirota* remains to be resolved, our proposals of the genus “*Ca.* Manganitrophus,” family “*Ca.* Manganitrophaceae,” and order “*Ca.* Troglogloeales” are supported by both 16S rRNA gene and genome phylogenetic approaches and reveal members of a novel marine genus that possibly oxidize Mn lithotrophically.

### Genome comparison streamlines the hypothesized genes for Mn-oxidizing lithotrophy.

**FIG 3:**
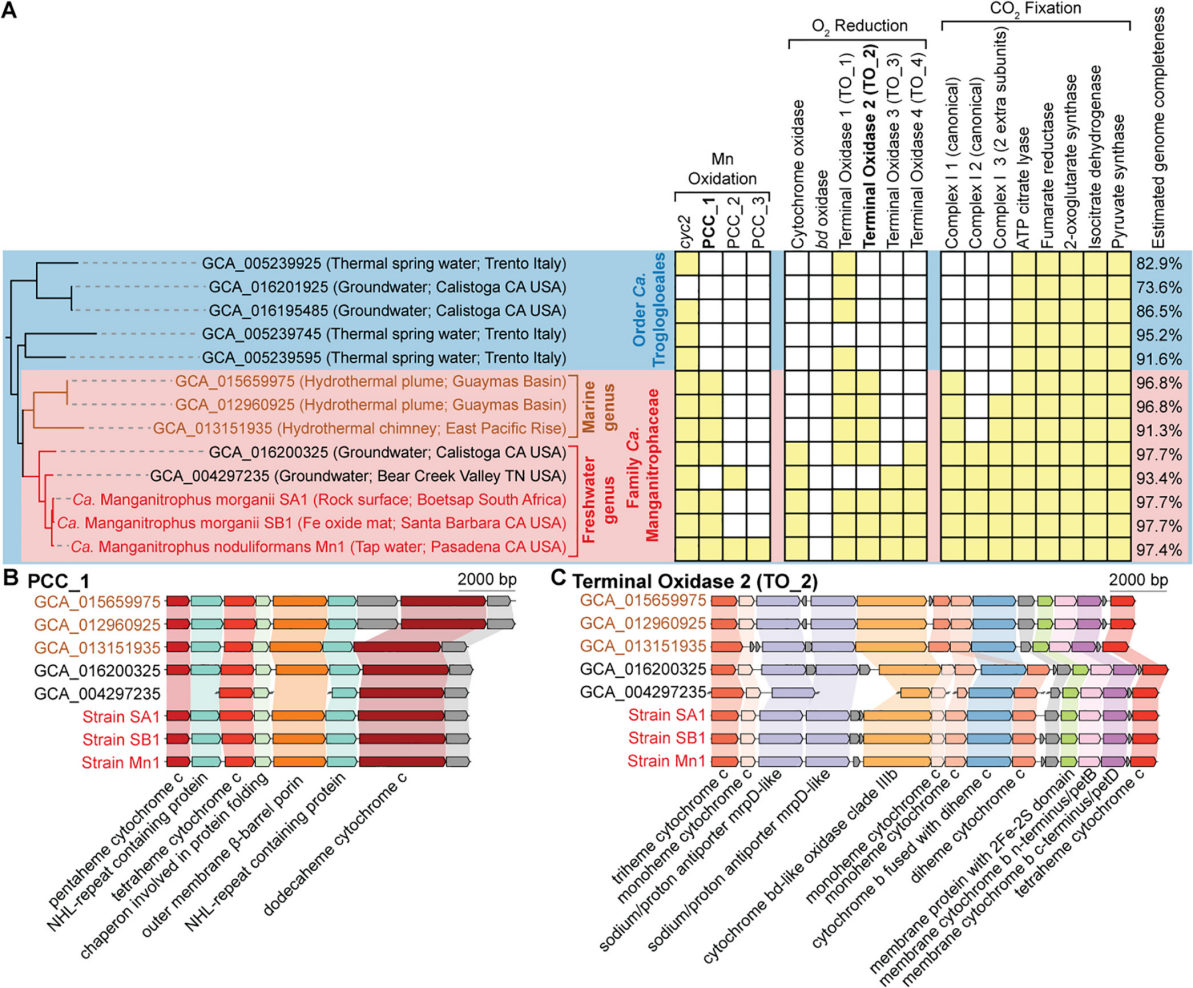
Metabolic genes and gene clusters of interest in metagenome-assembled genomes representing the order “*Candidatus* Troglogloeales.” (A) The multilocus protein phylogram and the presence (yellow filled square) or absence (empty square) of genes and gene clusters of interest in the corresponding genomes. Putative functional assignments are proposed above the gene and gene cluster names. The phylogram (left) is extracted from Fig. 2. (B and C) Comparison of gene clusters of porin cytochrome *c* 1 (PCC_1) (B) and terminal oxidase 2 (TO_2) (C), both restricted to the family “*Candidatus* Manganitrophaceae.” Members of the freshwater genus “*Candidatus* Manganitrophus” share similar gene arrangements, which differ from those representing the *Candidatus* marine genus (in brown).

We next compared the MAGs of members of the family “*Ca.* Manganitrophaceae” to understand which genes might be candidates essential for Mn oxidation and whether these are found in representatives of the marine genus or other members in the phylum. Four routes for Mn oxidation and electron uptake had been previously hypothesized in strain Mn1, including a fused cytochrome-porin protein with a single heme *c* (Cyc2) and three different porin-dodecaheme cytochrome *c* (PCC) complexes (11). Cyc2 homologs are not only identified in the majority of “*Ca.* Troglogloeales” (Fig. 3A) but also in other members of the phylum, including characterized clades such as acidophilic, iron-oxidizing *Leptospirilla* and nitrite- or ammonia-oxidizing *Nitrospirales* (29, 30). Of the 3 PCCs in strain Mn1, only PCC_1 was found in the strains SA1 and SB1 (Fig. 3A). PCC_1 was also identified in other MAGs in both marine and freshwater genera of “*Ca.* Manganitrophaceae” but not in the extant MAGs and genomes of *Nitrospirota* species falling outside this family. These results point to PCC_1, possibly together with Cyc2, as being central to chemolithotrophic Mn oxidation by “*Ca.* Manganitrophaceae.”

Not all previously proposed membrane complexes by which strain Mn1 might reduce oxygen and conserve energy were shared by all “*Ca.* Manganitrophaceae” members. A canonical complex IV (*cbb3*-type cytochrome *c* oxidase) was identified for all members of the freshwater genus but not for any in the marine genus (Fig. 3A). Strain Mn1 had multiple genes for noncanonical cytochrome *bd*-like proteins that fall within gene clusters of terminal oxidase (TO) complexes (11). TO_1, a well-discussed terminal oxidase found in other *Nitrospirota* (31, 32), was also found in the majority of “*Ca.* Troglogloeales” (Fig. 3A). In contrast, complex TO_2, with its unusual two ion-pumping MrpD-like subunits that might be coupled to the generation or dissipation of a motive force (Fig. 3C), was restricted to “*Ca.* Manganitrophaceae” and remains a candidate for involvement in Mn(II) oxidation-dependent metabolism among these organisms. Complexes TO_3 and TO_4 were restricted to members of the freshwater genus only (Fig. 3A). As was the case for strain Mn1, a canonical cytochrome *bd* oxidase (hypothesized to be important to Fe-oxidizing, acidophilic *Leptospirilla* [33]) was not observed in any of the analyzed “*Ca.* Troglogloeales” (Fig. 3A).

### Autotrophic anabolism predicted for the Mn-oxidizing *Nitrospirota*.

Strain Mn1 was shown to be capable of CO_2_ fixation and autotrophic growth using Mn(II) as its electron donor (11). The majority of “*Ca.* Troglogloeales” and all “*Ca.* Manganitrophaceae” analyzed here encode complete gene sets for the reverse tricarboxylic acid (rTCA) cycle (Fig. 3A); members of the freshwater genus encode a class II fumarate hydratase, whereas those of the marine genus encode a class I form. A key gluconeogenic pathway gene (fructose-biphosphate aldolase), absent from strain Mn1 (11), was also absent from the majority of “*Ca.* Troglogloeales” (save for two of the MAGs not central to this study, NCBI assembly accessions GCA_004297235 and GCA_013151935). Only 1 of 5 candidate pyruvate dehydrogenase genes in the genome of strain Mn1 is shared among all members of the “*Ca.* Manganitrophaceae.”

**FIG 4:**
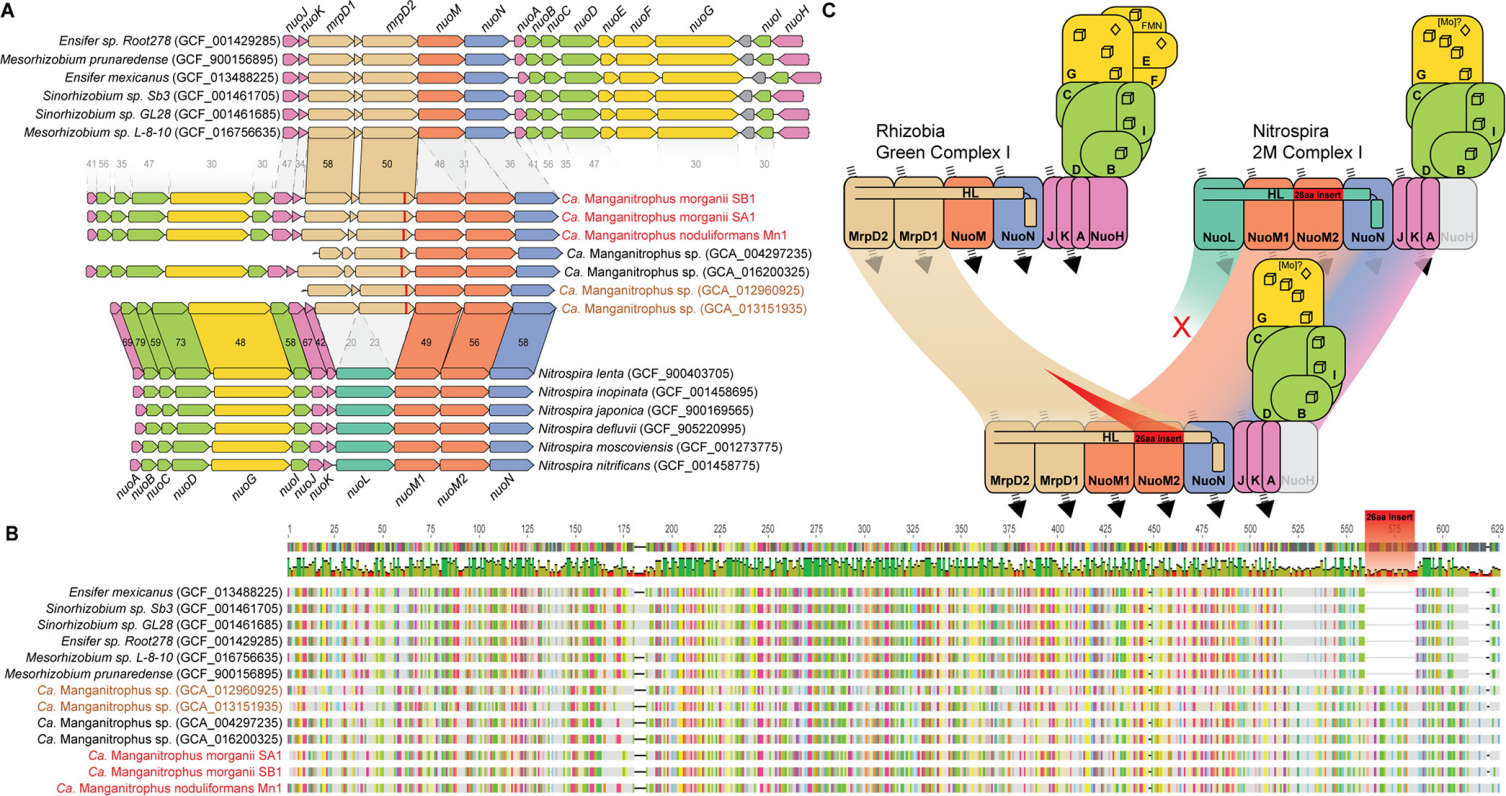
Highly unusual complex I (Complex_I_3) with two extra pumping subunits unique to “*Candidatus* Manganitrophaceae.” (A) Comparison of gene clusters of unusual complex I with extra pumping subunits in “*Ca.* Manganitrophus” (middle) with their closest homologs in rhizobia (top) and *Nitrospira* (bottom). Homologs are connected between the 3 different organism clades, with values representing the average amino acid identities of proteins between the clades. NCBI accession numbers for the genome assemblies are included in parentheses in the organism names. (B) Sequence alignment of MrpD2 in Complex_I_3 in “*Ca.* Manganitrophaceae” reveals a 26-amino-acid insert (red) compared to their closest homologs in rhizobia. (C) Sequence comparisons reveal that Complex_I_3 in “*Ca.* Manganitrophaceae” is likely a hybrid between the green complex I in rhizobia and the 2M complex I in *Nitrospira*. Given their sequence similarities, the two MrpDs in Complex_I_3 could be derived from rhizobia, whereas the other components in Complex_I_3 could be derived from *Nitrospira*. The 1 to 2 extra pumping subunits in these unusual complex I could enable translocation of a total of 5 to 6 protons or ions (as indicated by dashed arrows) compared to the 4 protons translocated by the canonical complex I.

The rTCA cycle requires low-potential electrons in the form of both NAD(P)H and reduced ferredoxin (34), yet Mn(II)-derived electrons are considered to be of too high potential to be able to generate such reducing power. Reverse electron transport, by running complex I in reverse, has been shown or postulated previously (11, 35, 36). In that regard, the genome of strain Mn1 is unusual in that it encodes 3 different complex I gene clusters (11). Two of these (Complex_I_1 and Complex_I_2) were similar to the canonical forms in both gene content and order (Fig. S2); the first was shared by all members of “*Ca.* Manganitrophaceae,” the second only by members of the freshwater genus (Fig. 3A). Strikingly, while the third and highly unusual form (Complex_I_3) was encoded by all “*Ca.* Manganitrophaceae” (except for one MAG), this complex was not found in any other member of the phylum analyzed here (Fig. 3A). Complex_I_3 is unique to the known biological world because it encodes two additional ion-pumping subunits for a total of five (Fig. 4). Other unusual examples known to biology include those with a fourth ion-pumping subunit: (i) *Nitrospira* 2M complex I (35), encoded by an additional NuoM, and (ii) green complex I in certain rhizobia, wherein 2 MrpD-like subunits replace the single canonical NuoL (37). Here, sequence analyses of the Complex_I_3 subunits encoded by “*Ca.* Manganitrophaceae” revealed a hybrid structure between the aforementioned: the two MrpD-like subunits were most closely related to those of the rhizobial green complex I, whereas the two NuoM and one NuoN subunits were most closely related to those in the *Nitrospira* 2M complex I (Fig. 4A). Sequence alignments of the MrpD2 subunits encoded by “*Ca.* Manganitrophaceae” revealed a conserved 26-amino-acid insertion within the C-terminal amphipathic helix (HL) that was not observed in the related MrpD subunits found in the rhizobial green complex I (Fig. 4B). An insertion of similar length and position was previously identified in NuoL of *Nitrospira* 2M complex I and postulated to accommodate interactions across the four (instead of the standard three) ion-pumping subunits (35). Curiously, in “*Ca.* Manganitrophaceae,” such unusual insertions were not unique to MrpD2 of Complex_I_3, as they were also found in the NuoL subunit of Complex_I_1 shared by all “*Ca.* Manganitrophaceae” as well as in Complex_I_2 shared by the freshwater forms (Fig. S2). The multiple versions of complex I found in each of these genomes are either already able to accommodate additional ion-pumping subunits or are in evolutionary transition toward or away from having such a capacity (Fig. 4C).

10.1128/mbio.03421-21.3 FIG S2 Comparison of two canonical complex I in “*Candidatus* Manganitrophaceae” and their closest homologs reveal insertions in NuoL subunits of “*Ca.* Manganitrophaceae.” (A) Comparison of gene clusters and sequence alignments of Complex_I_2. NCBI accession for NuoL of Complex_I_2 in “*Ca.* Manganitrophus” noduliformans Mn1 is WP_168059645. (B) Comparison of gene clusters and sequence alignments of Complex_I_1. NCBI accession for NuoL of Complex_I_1 in “*Ca.* Manganitrophus” noduliformans Mn1 is WP_168062695. Download FIG S2, JPG file, 2.3 MB. Copyright © 2022 Yu et al. 2022 Yu et al. https://creativecommons.org/licenses/by/4.0/ This content is distributed under the terms of the Creative Commons Attribution 4.0 International license.

### Core genome of “*Ca.* Manganitrophaceae” in marine and freshwater environments.

*De novo* gene clustering revealed that 8 analyzed members of “*Ca.* Manganitrophaceae” shared a total of 895 gene clusters, which included the above-mentioned Cyc2, PCC_1, TO_1, TO_2, Complex_I_1, and Complex_I_3 (Table S6). Several other shared genes and pathways appear noteworthy: assimilatory sulfate reduction (*sat*, *aprA-B*, *aSir*), cytochrome *c* biogenesis, heme exporters, 2 multicopper oxidases, and type IV pilus assembly. These confirm the basis for the ability of the cultivated strains to use sulfate as an anabolic sulfur source, make cytochrome *c* for anabolism and catabolism, and suggest the potential for twitching surface motility. Notably missing among the shared genes were those for the carbon monoxide dehydrogenase complex that had been observed to be highly expressed (95th percentile) during Mn(II)-dependent growth by strain Mn1 (11). Together, our comparative genomic analyses shed light on common gene sets of Mn-oxidizing chemolithoautotrophs in both marine and freshwater environments.

10.1128/mbio.03421-21.9 TABLE S6 *De novo* gene clusters of “*Candidatus* Manganitrophaceae.” Download Table S6, XLSX file, 1.3 MB. Copyright © 2022 Yu et al. 2022 Yu et al. https://creativecommons.org/licenses/by/4.0/ This content is distributed under the terms of the Creative Commons Attribution 4.0 International license.

## DISCUSSION

**FIG 5:**
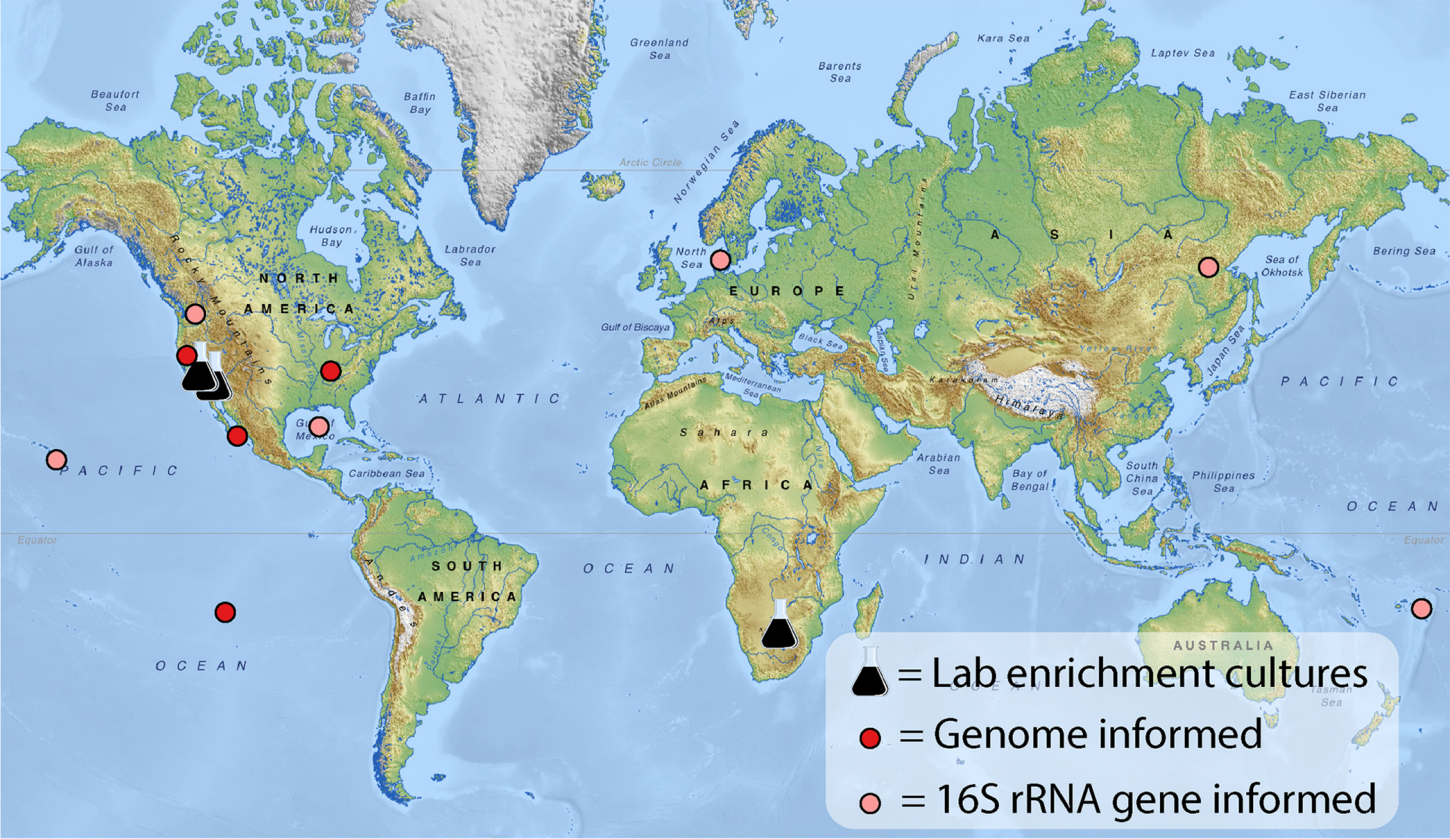
Distribution of cultures, metagenome-assembled genomes, and phylotypes representing “*Candidatus* Manganitrophaceae” implies their worldwide reach in freshwater and marine environments. Freely available map (https://mapswire.com/world/physical-maps/), modified as permitted under the Creative Commons Attribution 4.0 International License.

Cultivation of novel microorganisms with previously undemonstrated physiologies remains a key cornerstone to our expanding understanding of the metabolic potential of the largely uncultured microbial diversity in nature (38, 39). Aerobic, Mn(II)-oxidizing chemolithoautotrophs were long theorized but only recently demonstrated to exist *in vitro* in a bacterial coculture (11). The majority member was a distinct member of the phylum *Nitrospirota*, “*Ca.* Manganitrophus noduliformans” strain Mn1, and only distantly related to any other cultivated biota (11). Curiously, the initial enrichment of Mn(II)-oxidizing chemolithoautotrophs from Caltech’s campus tap water was unintentional (11). Here, cultivation attempts were intentionally initiated with the specific goal of successfully establishing new Mn-oxidizing enrichment cultures. These attempts were successful using a medium formulation refined during the course of the earlier study using inocula obtained from two different continents and hemispheres. Community analyses on these two new enrichment cultures revealed that the most abundant microorganisms in each were closely related to, but of a different species than, “*Ca.* M. noduliformans” strain Mn1. The enrichment cultures also harbored a diversity of taxa varying in their relative abundances and identities (Fig. 1). The results support the notion that members of the genus “*Ca.* Manganitrophus” are playing a key if not the central role in chemolithoautotrophic Mn(II) oxidation in the laboratory cultures examined. The results also suggest that “*Ca.* Manganitrophus” does not require an obligate partnership with *R. lithotrophicus* (the second species present in the previously described coculture [11]), leaving open the possibility that its eventual clonal isolation is possible. The phylogenomic analyses here also predict an assemblage of a marine genus within the family “*Ca.* Manganitrophaceae” that may also carry out this mode of chemolithoautotrophy (Fig. 2 to 4). However, our analyses do not exclude other members in *Nitrospirota* carrying out Mn(II) lithotrophy using a different mechanism than that we hypothesized for “*Ca.* Manganitrophacae.” With the increasing evidence that the “*Ca.* Manganitrophaceae” are distributed globally across marine and freshwater biomes (Fig. 5) taken together with the reported prevalence of Mn and Mn-reducing microorganisms in the environment (14, 40), chemolithoautotrophic Mn oxidation becomes particularly important to reaching a better understanding of the redox biogeochemical cycle for manganese.

**FIG 6:**
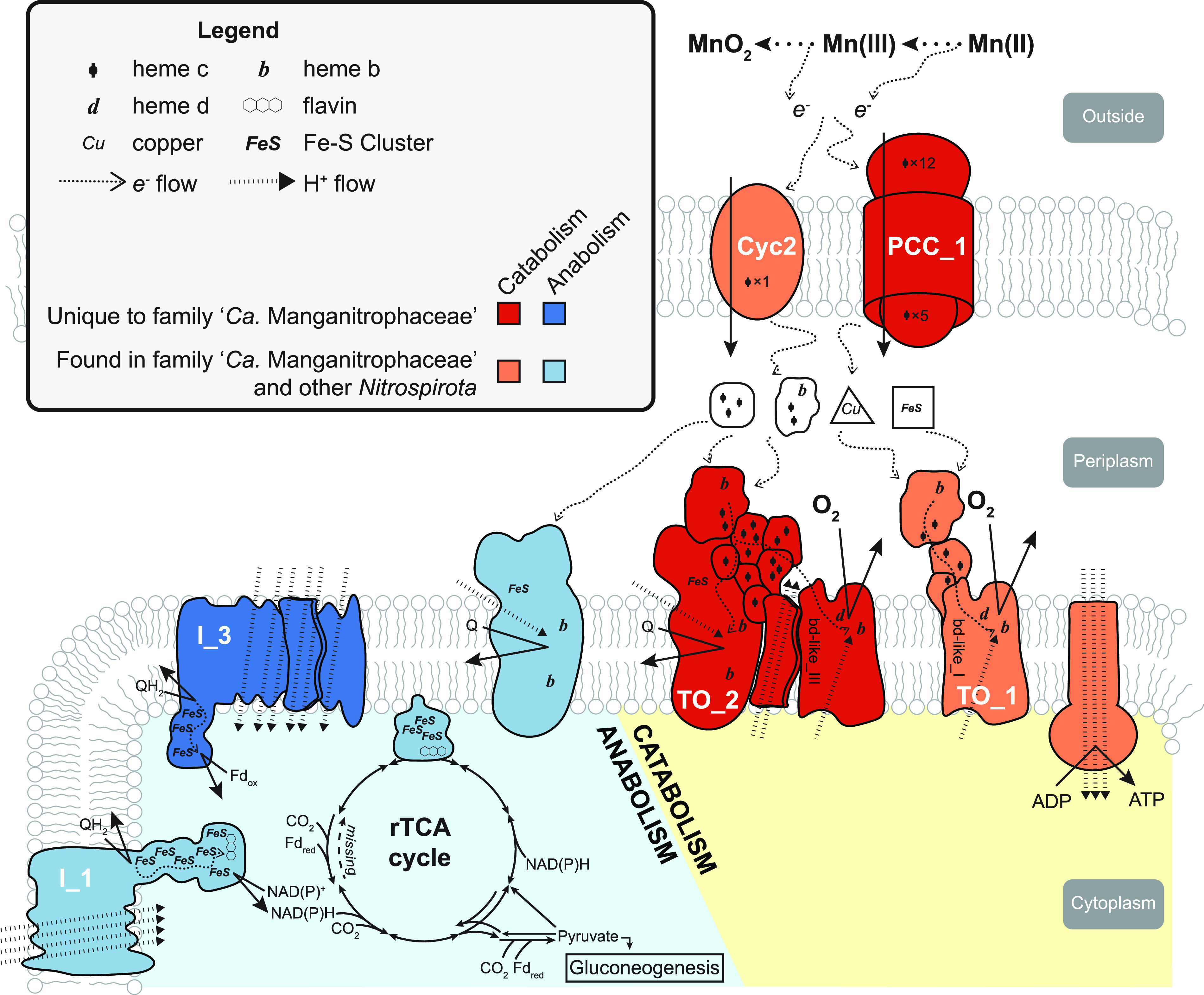
Key proteins and complexes putatively facilitating manganese chemolithoautotrophy in “*Candidatus* Manganitrophaceae,” as deduced from representative genomes.

By comparing metagenome-assembled genomes of the 3 cultivated “*Ca.* Manganitrophus” strains and related but uncultivated organisms available in public genome databases, our results narrow down the list of genes in “*Ca.* Manganitrophaceae” that may underlie Mn(II) oxidation-driven chemolithoautotrophy. Unique to “*Ca.* Manganitrophaceae” among all *Nitrospirota*, and perhaps across all of the biological world that has been analyzed, were PCC_1, as a candidate for being the initial electron acceptor during Mn oxidation, TO_2, as a candidate respiratory complex for productively coupling the electrons from Mn(II) oxidation to oxygen reduction and energy conservation (Fig. 3 and 6), and Complex_I_3, as a candidate complex catalyzing reverse electron transport to generate low-potential reducing power from quinones during carbon fixation (Fig. 4 and 6).

While not unique to “*Ca.* Manganitrophaceae,” the identification of Cyc2 and TO_1 in the majority of the family members (Fig. 3A and 4B), together with their comparable or even higher expression than that of PCC_1 and TO_2, respectively, in strain Mn1 (11), suggests that these two complexes are involved in Mn lithotrophy. Cyc2 is a fused cytochrome-porin protein with a single heme *c*, whereas porin cytochrome *c* (PCC) are larger complexes composed of a beta-barrel outer membrane protein and at least one multiheme cytochrome *c* (41–43). Variants of both are better understood in acidophilic and circumneutral pH Fe(II) oxidizers. Key predicted structural differences between the two include an inner placement of heme *c* within a smaller porin size for Cyc2, suggesting that Cyc2 only reacts with dissolved Fe^2+^ species (29), whereas PCC variants have been suggested to react with both soluble and insoluble forms of Fe(II). In the case of Mn(II), the oxidation is thought most likely to proceed via two sequential one-electron oxidation steps (44). In that case, Cyc2 and PCC_1 might serve to react with different species of Mn(II) [e.g., soluble Mn(H_2_O)_6_^2+^, soluble or insoluble MnCO_3_, or Mn(HCO_3_)_2_] or different oxidations of Mn(II) [e.g., Mn(II) versus Mn(III)]. Employing Cyc2 and PCC_1 would differ from well-studied nonlithotrophic heterotrophs that catalyze direct Mn(II) oxidations with O_2_ or reactive oxygen species, e.g., via multicopper oxidase (MCO) or heme peroxidase homologs (45–47), involving mechanisms without a clear path for free energy conservation. While members of “*Ca.* Manganitrophaceae” all encode two novel MCOs each (Table S6), for these to be involved in Mn(II) lithotrophy, Mn(II)-derived electrons would need to be transferred to a periplasmic electron carrier, such as cytochrome *c*, rather than directly to oxygen (48).

Instead of using canonical cytochrome *c* oxidases for oxygen respiration, “*Ca.* Manganitrophaceae” appear to rely on poorly characterized terminal oxidase (TO) complexes (Fig. 6). In strain Mn1, 4 TO complexes all contained cytochrome *bd*-like proteins, but other deduced protein components differed between them (11). TO_1 contained a periplasmic cytochrome *b* that may receive electrons from the periplasm, whereas TO_3 and TO_4 contained complex III or alternative complex III-like components that may interact with the quinone pool (11). From the analyses here, TO_2 stood out. It was found to be unique to and shared among all examined “*Ca.* Manganitrophaceae,” and its deduced structure included both a periplasm-accessible and membrane-embedded cytochrome *b* that might serve to receive electrons from a periplasmic carrier and to transfer them to the quinone pool (Fig. 3C and 4B). In theory, TO_2 might even bifurcate Mn(II)-derived electrons (on average a E°′ of +466 mV) to reduce higher potential oxygen (E°′ of +818 mV [21] via its *bd*-like oxidase) concomitant with lower potential quinones (E°′ of ∼+113 mV [21] via its membrane cytochrome *b*). If so, a role of the 2 noncanonically placed MrpD-like subunits in this complex could be dissipation of ion motive force to drive the otherwise endergonic reduction of quinones (Fig. 6), which in turn could serve as substrates for reverse electron transport by the unusual complex I_3, i.e., to generate low-potential reductant for rTCA-mediated carbon fixation (35). Our analyses of Complex_I_3 examining subunit similarities, gene clustering, and the presence of specific insertions (Fig. 4A and B) suggest an evolutionary hybridization wherein the MrpD subunits of a rhizobium-like green complex I replaced the NuoL of a *Nitrospira*-like 2M complex I, with an additional HL extension needed in MrpD2 of Complex_I_3 to accommodate the second NuoM (Fig. 4C). If run in reverse, this highly unusual complex, having a total of 5 ion-pumping subunits, might drive the otherwise endergonic transfer of electrons from the reduced quinone pool to a carrier having a lower reduction potential than that of NADH, such as ferredoxin, required for the rTCA cycle (Fig. 6). That is, the complex could serve to dissipate the motive force built up during Mn(II) lithotrophy by coupling the inward flow of 6 protons or sodium ions with the otherwise endergonic reduction of a ferredoxin, using a quinol (Fig. 4C and 5B). The additional pumping subunit in “*Ca.* Manganitrophaceae” compared to *Nitrospira* species suggests that the utilization of Mn(II)-derived electrons for carbon fixation via the reverse TCA cycle poses an added bioenergetic challenge compared to the use of other high-potential electron donors, such as nitrite or ammonia.

Based on our phylogenomic analyses, a set of shared, unique complexes in “*Ca.* Manganitrophaceae,” namely, PCC_1, TO_2, and Complex_I_3, become prime targets for future physiological and biochemical examination in efforts to better understand the cellular machinery enabling Mn(II)-dependent chemolithoautotrophy. Much of our proposed routes of the oxidation of Mn(II) to Mn(III) and Mn(IV) are in large part informed by existing knowledge on the single electron oxidation of Fe(II) to Fe(III). Fe(II) oxidizers have been found in diverse marine and freshwater environments (49, 50), as is now the case for cultivated and demonstrated as well as uncultivated and putative Mn(II) oxidizers in “*Ca.* Manganitrophaceae” (Fig. 5). Taxonomically, Fe(II) oxidizers have been identified in several phyla of bacteria and archaea (49, 50) and can be acidophiles or neutrophiles, mesophiles or thermophiles, phototrophs or chemotrophs, heterotrophs or autotrophs, and aerobes or anaerobes (49, 50). If such extends to the biology of energetic Mn(II) oxidation, the results gleaned here from the cultivation and phylogenomics of “*Ca.* Manganitrophaceae” may be only the first glimpse into the full diversity of microorganisms capable of coupling Mn(II) oxidation to growth.

## MATERIALS AND METHODS

### Cultivation.

The enrichment procedure and manganese carbonate medium composition (using 1 mM nitrate or ammonia as the N source, as noted) were described previously (11). Unless stated otherwise, culturing was performed in 10 mL of medium in 18-mm culture tubes. Cultures were transferred (10%, vol/vol) when laboratory prepared MnCO_3_ (light pink or tan color) was completely converted to Mn oxide (dark or black color).

The South Africa inoculum was collected in June 2017 from a rock surface near a pond by a road on an exposed outcrop of the Reivilo Formation (lat −27.964167, long 24.454183; elevation, 1,107 m) near Boetsap, Northern Cape, South Africa. The rock was coated with a black material of a texture between slime and moss. A thin, laminated green mat was observed underlying the black material. The black material reacted to leucoberbelin blue dye, indicating the presence of manganese oxides. A mixture of the black and green material was sampled using an ethanol-sterilized spatula into a sterile 15-mL tube and stored at room temperature until inoculation. The cultures were initiated in medium with 1 mM ammonia and incubated at 28.5°C. Later, some were transferred to medium with 1 mM nitrate and/or incubated at 32°C.

The Santa Barbara inoculum was collected in November 2018 from an iron oxide mat surrounded by reeds at the outflow of a rusted iron pipe (lat 34.417944, long −119.741130) along the side of a road in Santa Barbara, CA, USA. The iron oxide mat was fluffy with a typical dark orange color. The mat was collected in a glass jar and stored at room temperature until inoculation. The enrichment cultures were incubated at 28.5°C, and later some were transferred to incubation at 32°C, all in the basal MnCO_3_ medium with 1 mM nitrate. The initial enrichment was transferred 5 times to confirm Mn-oxidizing activity and refine community composition prior to community and metagenomic analysis.

### Community analysis using 16S rRNA gene amplicon sequencing.

Mn oxides were harvested from stationary-phase enrichment cultures: 2 mL of culture containing ca. 0.15 g of Mn oxide nodules was sampled into a 2-mL Eppendorf tube and centrifuged at 8,000 × *g* for 3 min at room temperature. After carefully removing the supernatant by pipetting, DNA was immediately extracted from the pellets using the DNeasy PowerSoil kit (Qiagen, Valencia, CA, USA) by following the manufacturer’s instructions, with the bead beating option using FastPrep FP120 (Thermo Electron Corporation, Milford, MA, USA) at setting 5.5 for 45 s instead of the 10-min vortex step. DNA concentration was quantified using a Qubit double-stranded DNA (dsDNA) high-sensitivity assay kit (Thermo Fisher Scientific, Waltham, MA, USA).

For 16S rRNA gene amplicon sequencing, the V4-V5 region of the 16S rRNA gene was amplified from the DNA extracts using archaeal/bacterial primers with Illumina (San Diego, CA, USA) adapters on the 5′ end (515F, 5′-TCGTCGGCAGCGTCAGATGTGTATAAGAGACAGGTGYCAGCMGCCGCGGTAA-3′; 926R, 5′-GTCTCGTGGGCTCGGAGATGTGTATAAGAGACAGCCGYCAATTYMTTTRAGTTT-3′). Duplicate PCRs were pooled, barcoded, purified, quantified, and sequenced on Illumina’s MiSeq platform with 250-bp paired-end sequencing as previously described (11). Raw reads with a >1-bp mismatch to the expected barcodes were discarded, and indexes and adapters were removed using MiSeq Recorder software (Illumina). The reads then were processed using QIIME2 release 2020.11 (51). Briefly, forward and reverse reads were denoised using DADA2 (52) by truncating at positions 200 and 240, respectively, leaving 28-bp overlaps. Read pairs were merged and dereplicated and chimera removed with the “pooled” setting using DADA2 (52). Taxonomic assignments for the resulting amplicon sequencing variants (ASVs) used a pretrained naive Bayes classifier on the full-length 16S rRNA genes in the SILVA 138 SSURef NR99 database (53, 54). ASVs assigned to the same level 7 taxonomy were combined, and those assigned to mitochondria or chloroplast or without taxonomy assignments were removed using the –p-exclude mitochondria,chloroplast,“Bacteria;Other;Other;Other;Other;Other,”“Unassigned;Other;Other;Other;Other;Other” setting.

### Metagenomics.

Purified genomic DNA samples (2 to 50 ng) were fragmented to the average size of 600 bp via use of a Qsonica Q800R sonicator (power, 20%; pulse, 15 s on/15 s off; sonication time, 3 min). Libraries were constructed using the NEBNext Ultra II DNA library prep kit (New England Biolabs, Ipswich, MA) by following the manufacturer’s instructions (Novogene Corporation, Inc., Sacramento, CA, USA). Briefly, fragmented DNA was end-repaired by incubating the samples with an enzyme cocktail for 30 min at 20°C, followed by a second incubation for 30 min at 65°C. During end repair, the 5′ ends of the DNA fragments are phosphorylated and a 3′ A base is added through treatment with Klenow fragment (3′ to 5′ exo minus) and dATP. The protruding 3′ A base was then used for ligation with the NEBNext multiplex oligonucleotides for Illumina (New England Biolabs), which have a single 3′ overhanging T base and a hairpin structure. Following ligation, adapters were converted to the Y shape by treatment with USER enzyme, and DNA fragments were size selected using Agencourt AMPure XP beads (Beckman Coulter, Indianapolis, IN, USA) to generate fragment sizes between 500 and 700 bp. Adaptor-ligated DNA was PCR amplified with 9 to 12 cycles depending on the input amount, followed by AMPure XP bead clean-up. Libraries were quantified with a Qubit dsDNA HS kit (Thermo Fisher Scientific), and the size distribution was confirmed with high-sensitivity DNA Tapestation assay (Agilent Technologies, Santa Clara, CA, USA). Sequencing was performed on the HiSeq platform (Illumina) with paired 150-bp reads by following the manufacturer’s instructions (Novogene). Base calls were performed with RTA v1.18.64 followed by conversion to FASTQ with bcl2fastq v1.8.4 (Illumina). In addition, reads that did not pass the Illumina chastity filter, as identified by the Y flag in their fastq headers, were discarded.

The resulting reads were uploaded to the KBase platform (55), trimmed using Trimmomatic v0.36 (56) with default settings and adaptor clipping profile Truseq3-PE, and assembled using Spades v3.11.1 (57) with default settings for the standard data set. Manual binning and scaffolding were performed using mmgenome v0.7.179 based on differential coverage and GC content of different metagenomes to generate the MAG for the most abundant organism. MAGs were annotated using the Rapid Annotations using Subsystems Technology (RAST) (58–60) and NCBI Prokaryotic Genome Annotation (61) pipelines. Average nucleotide identities and reciprocal mapping of MAGs were done using fastANI v1.32 (24). Average amino acid identities were done using enve-omics tool AAI calculator (26). *De novo* gene clustering was done using anvio v7 with default parameters (62). Comparison of complex I gene clusters was done using protein-protein BLAST with default parameters (63) to the RefSeq Select protein database (64). Alignment of complex I gene sequences was done using MUSCLE v3.8.1551 with default parameters (65).

### Phylogenetic analyses.

For genome phylogeny, 433 publicly available genome assemblies in the NCBI Assembly Database (61) fell within the phylum *Nitrospirae* (taxonomy identifier [ID] 40117) (66), and 6 publicly available genomes in the genomic catalog of Earth’s microbiome data set (67) fell within the phylum *Nitrospirota* under the headings Nitrospirota and Nitrospirota_A (27) and were analyzed (as of 30 March 2021). For 16S rRNA gene phylogeny, 16s rRNA genes from the MAGs of *Nitrospirota* from the enrichment metagenomes, as well as the genome assemblies, were retrieved using CheckM v1.1.2 (68) ssu_finder utility. Sequences less than 900 bp were excluded. The 16S rRNA gene sequences were aligned using SINA v1.2.11 (69) and imported into SILVA Ref Database release 138.1 (53). A total of 104 16S rRNA gene sequences, including 5 different outgroup sequences (Desulfovibrio vulgaris, Ramlibacter tataouinensis TTB310, Nitrospina gracilis 3/211, Acidobacterium capsulatum, and “*Candidatus* Methylomirabilis oxyfera”), with 1,508 nucleotide positions, were exported with the bacterial filter excluding columns with mostly gaps from ARB software v6.0.2 (70). Bayesian phylogenetic trees were constructed using MrBayes v3.2.7 (71), with the evolutionary model set to GTR + I + gamma, burn-in set to 25%, and stop value set to 0.01, and edited in iTOL v6 (72). For concatenated multilocus protein phylogeny, marker proteins from 104 genomes including the same 5 outgroup species were identified and aligned using a set of 120 ubiquitous single-copy bacterial proteins in GTDB v0.2.2 (27). The protein alignment was filtered using default parameters in GTDB v0.2.2 (27) (the full alignment of 34,744 columns from 120 protein markers was evenly subsampled with a maximum of 42 columns retained per protein; a column was retained only when the column was in at least 50% of the sequences and contained at least 25% and at most 95% of one amino acid). The resulting alignment with 5,040 amino acid positions was used to construct the multilocus protein phylogeny using MrBayes v3.2.7 (71) as described above, except the evolutionary model was set to invgamma and a mixed amino acid model.

### Data availability.

The partial 16S rRNA gene amplicon sequences of enrichment cultures and metagenome-assembled genomes of “*Candidatus* Manganitrophus morganii” strains SA1 and SB1 have been deposited with the National Center for Biotechnology Information (NCBI) under BioProject no. PRJNA776098.
