# Supplementary material for: Comparative Genomics on Cultivated and Uncultivated Freshwater and Marine “Candidatus Manganitrophaceae” Species Implies Their Worldwide Reach in Manganese Chemolithoautotrophy
Source: mBio. 2022 Mar 14;13(2):e03421-21. doi: 10.1128/mbio.03421-21 (PMC9040806; doi:10.1128/mbio.03421-21)
Supplement: TEXT S1 [file mbio.03421-21-s0001.docx]

**Supplemental Text - Taxonomic Description of “*Candidatus* Manganitrophus morganii”, sp. nov.**

Description of “*Candidatus* Manganitrophus morganii”, sp. nov.

*Candidatus* “Manganitrophus morganii” [mor.gan’i.i]. N.L. gen. n. morganii, named after James J. Morgan, PhD (1932–2020), the aquatic chemist, textbook author, Stockholm Water Prize recipient, and Professor of Environmental Engineering Science at Caltech, whose long career focused on the environmental chemistry of manganese.

Cultures exhibit aerobic, chemolithoautotrophic growth on manganese carbonate, utilizing the Mn(II) as sole energy source. Liquid cultures grown in manganese carbonate media generate manganese oxide nodules and concretions with which the cells are found associated. Colonies from single cells have not yet been observed to form in or on agar, or to grow in the absence of co-enriched strains of multiple other microbial species. By genomics, cells are predicted to employ the reverse tricarboxylic acid cycle during their autotrophic growth. Growth by Mn(II) oxidation has been observed to occur at circumneutral pH and at 28.5 °C. By genomics, cells are predicted to be able to assimilate sulfate, but unable to grow lithotrophically via the oxidation of either H_2_, carbon monoxide, ammonia, nitrite, or reduced sulfur substrates, or to grow anaerobically via denitrification or dissimilatory nitrate reduction. Based on phylogenetic reconstructions using 16S rRNA gene and/or protein sequences, the species affiliates within the proposed phylum *Nitrospirota* (*Nitrospirae*), is distinct from the genera comprising the classes *Nitrospiria* and *Leptospirillia*, and clusters with both cultivated and not yet cultivated members of the family ‘*Ca.* Manganitrophaceae’, specifically within the genus ‘*Ca.* Manganitrophus’. The species can be discriminated from the type strainand only other cultivated species representing the genus, ‘*Ca.* M. noduliformans’ Strain Mn1, on the basis of both gene content, an ANI score of <94.0, and an average amino acid identity of <93.3.

The type (meta)genome gene sequence of Strain SB1, the type for the candidatus species is: DDBJ/ENA/GenBank JAJHOH000000000^TS^. This were obtained from a multispecies enrichment culture initiated with a sample from an iron oxide mat surrounded by reeds at the outflow of a rusted iron pipe (34.417944, -119.741130) along the side of a road in Santa Barbara, California, USA. The (meta)genome of the type, resolved to a single molecule from other species in the mixed culture in which it dominated, is 4.29 Mbp in size and has a G+C content of 56.2 mol%.

A second distinct cultivar, ‘*Ca.* M. morganii’ Strain SA1, was obtained from a multispecies enrichment culture initiated with a sample of a biological layer covering a rock surface near a pond by a road on an exposed outcrop of the Reivilo Formation (-27.964167, 24.454183, elevation 1107 m) near Boetsap, Northern Cape, South Africa. The (meta)genome gene sequence of Strain SA1 is: DDBJ/ENA/GenBank JAJHOI000000000. The (meta)genome of the type, resolved from other species in the mixed culture in which it dominated, is 4.26 Mbp in size and has a G+C content of 56.1 mol%. Strain SA1 can be discriminated from ‘*Ca.* M. morganii’ Strain SB1, the only other cultivated strain and type representing the species, on the basis of gene content, an ANI score of 96.0, and an average amino acid identity of 95.3; as well as by the ability to grow at 32 °C, a temperature at which type Strain SB1 does not.
