# Supplementary figures and images for: Comparative Genomics on Cultivated and Uncultivated Freshwater and Marine “Candidatus Manganitrophaceae” Species Implies Their Worldwide Reach in Manganese Chemolithoautotrophy
Source: mBio. 2022 Mar 14;13(2):e03421-21. doi: 10.1128/mbio.03421-21 (PMC9040806; doi:10.1128/mbio.03421-21)

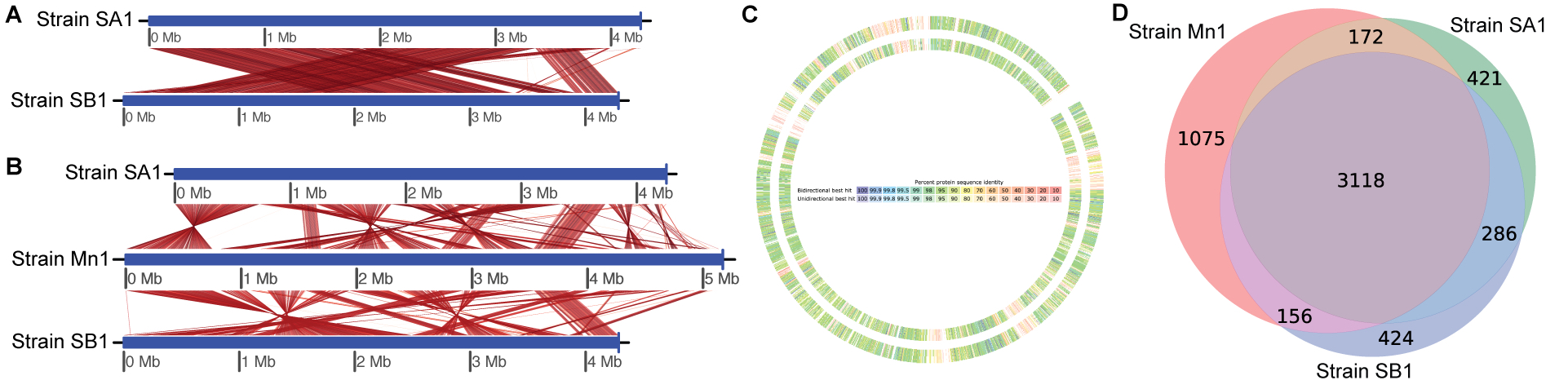

Supplement: FIG S1 [file mbio.03421-21-sf001.jpg]

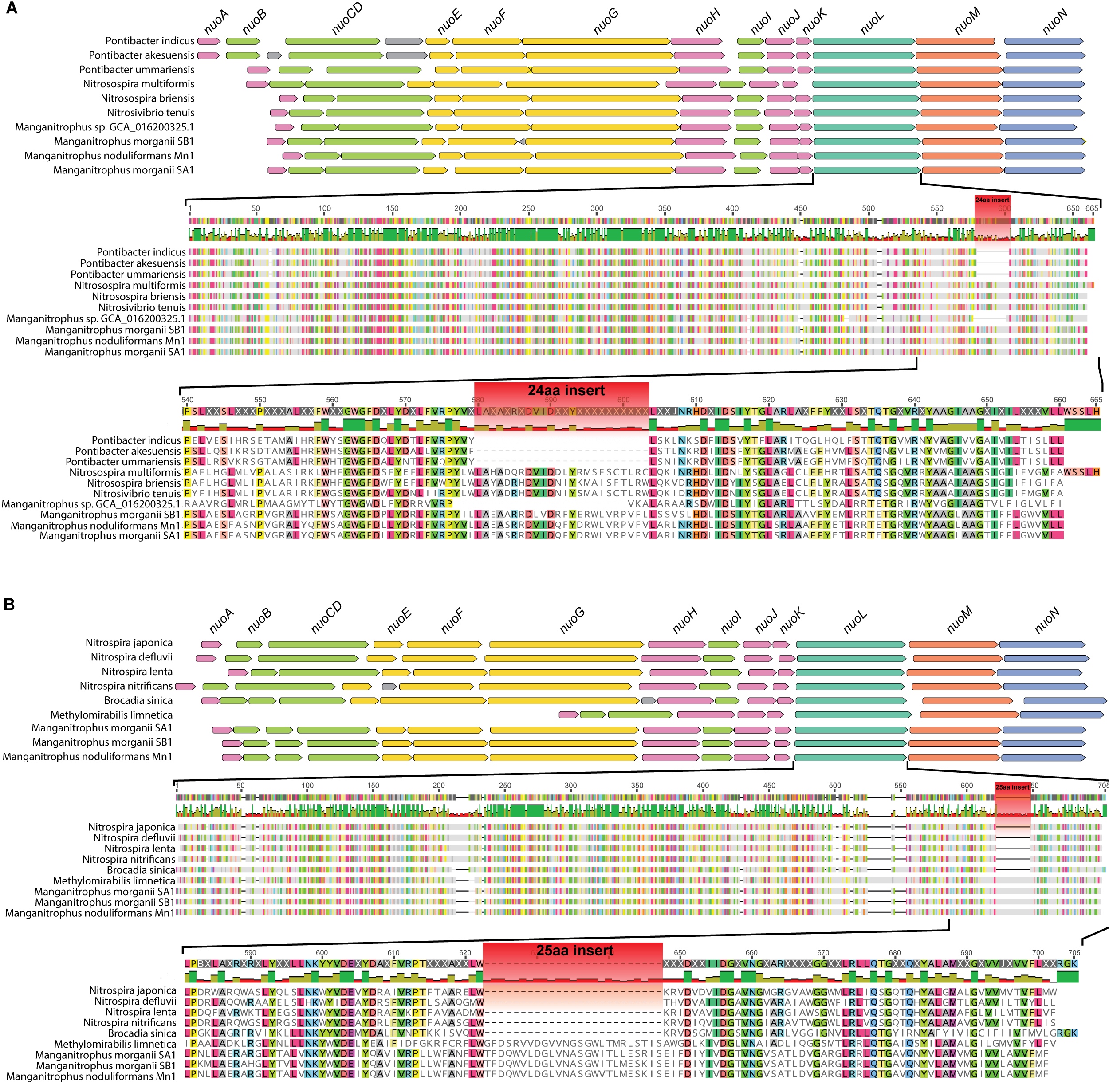

Supplement: FIG S2 [file mbio.03421-21-sf002.jpg]
