# Supplementary material for: Comparative Genomics on Cultivated and Uncultivated Freshwater and Marine “Candidatus Manganitrophaceae” Species Implies Their Worldwide Reach in Manganese Chemolithoautotrophy
Source: mBio. 2022 Mar 14;13(2):e03421-21. doi: 10.1128/mbio.03421-21 (PMC9040806; doi:10.1128/mbio.03421-21)
Supplement: TABLE S2 [file mbio.03421-21-st002.docx]

**Supplementary Table 2. Assembly and genome statistics of two new *Ca.* Manganitrophus morganii strains in enrichment cultures.**

| **Bin Name** | **Completeness** | **Contamination** | **Total length** | **# Contigs** | **GC** | **Coverage** |
| --- | --- | --- | --- | --- | --- | --- |
| **Strain SB1 (GCA_021651055)** | 97.67 | 3.64 | 4,287,287 bp | 1 | 56.20% | 436x |
| **Strain SA1**  **(GCA_021651075)** | 97.67 | 3.64 | 4,257,136 bp | 6 | 56.10% | 1024x |
