# Supplementary material for: Comparative Genomics on Cultivated and Uncultivated Freshwater and Marine “Candidatus Manganitrophaceae” Species Implies Their Worldwide Reach in Manganese Chemolithoautotrophy
Source: mBio. 2022 Mar 14;13(2):e03421-21. doi: 10.1128/mbio.03421-21 (PMC9040806; doi:10.1128/mbio.03421-21)
Supplement: TABLE S3 [file mbio.03421-21-st003.docx]

**Supplementary Table 3. Average nucleotide identity (ANI) comparison of genomes and metagenome-assembled genomes in the family *Candidatus* Manganitrophaceae.** ANI values less than 0.75 are unreliable and therefore given a value of 0 instead. The MAG IDs of the marine genus are in brown.

|  | **GCA_012960925** | **GCA_013151935** | **GCA_015659975** | **GCA_016200325** | **GCA_004297235** | **Strain Mn1** | **Strain SA1** | **Strain SB1** |
| --- | --- | --- | --- | --- | --- | --- | --- | --- |
| **GCA_012960925** | 1.000 | 0.000 | 1.000 | 0.000 | 0.000 | 0.000 | 0.000 | 0.000 |
| **GCA_013151935** | 0.000 | 1.000 | 0.000 | 0.000 | 0.000 | 0.000 | 0.000 | 0.000 |
| **GCA_015659975** | 1.000 | 0.000 | 1.000 | 0.000 | 0.000 | 0.000 | 0.000 | 0.000 |
| **GCA_016200325** | 0.000 | 0.000 | 0.000 | 1.000 | 0.792 | 0.789 | 0.791 | 0.792 |
| **GCA_004297235** | 0.000 | 0.000 | 0.000 | 0.793 | 1.000 | 0.821 | 0.819 | 0.822 |
| **Strain SA1** | 0.000 | 0.000 | 0.000 | 0.789 | 0.819 | 0.937 | 1.000 | 0.960 |
| **Strain Mn1** | 0.000 | 0.000 | 0.000 | 0.789 | 0.821 | 1.000 | 0.937 | 0.939 |
| **Strain SB1** | 0.000 | 0.000 | 0.000 | 0.792 | 0.822 | 0.940 | 0.960 | 1.000 |
