# Supplementary material for: Comparative Genomics on Cultivated and Uncultivated Freshwater and Marine “Candidatus Manganitrophaceae” Species Implies Their Worldwide Reach in Manganese Chemolithoautotrophy
Source: mBio. 2022 Mar 14;13(2):e03421-21. doi: 10.1128/mbio.03421-21 (PMC9040806; doi:10.1128/mbio.03421-21)
Supplement: TABLE S4 [file mbio.03421-21-st004.docx]

**Supplementary Table 4. Average amino acid identity comparison of genomes and metagenome-assembled genomes in the family *Candidatus* Manganitrophaceae.** The MAG IDs of the marine genus are in brown.

|  | **GCA_012960925** | **GCA_013151935** | **GCA_015659975** | **GCA_016200325** | **GCA_004297235** | **Strain SA1** | **Strain Mn1** | **Strain SB1** |
| --- | --- | --- | --- | --- | --- | --- | --- | --- |
| **GCA_012960925** | 1.000 | 0.603 | 1.000 | 0.611 | 0.618 | 0.623 | 0.615 | 0.621 |
| **GCA_013151935** | 0.603 | 1.000 | 0.606 | 0.560 | 0.564 | 0.566 | 0.559 | 0.565 |
| **GCA_015659975** | 1.000 | 0.606 | 1.000 | 0.614 | 0.620 | 0.626 | 0.619 | 0.623 |
| **GCA_016200325** | 0.611 | 0.560 | 0.614 | 1.000 | 0.768 | 0.749 | 0.746 | 0.753 |
| **GCA_004297235** | 0.618 | 0.564 | 0.620 | 0.768 | 1.000 | 0.809 | 0.809 | 0.817 |
| **Strain SA1** | 0.623 | 0.566 | 0.626 | 0.749 | 0.809 | 1.000 | 0.933 | 0.953 |
| **Strain Mn1** | 0.615 | 0.559 | 0.619 | 0.746 | 0.809 | 0.933 | 1.000 | 0.931 |
| **Strain SB1** | 0.621 | 0.565 | 0.623 | 0.753 | 0.817 | 0.953 | 0.931 | 1.000 |
