# Supplementary material for: Comparative Genomics on Cultivated and Uncultivated Freshwater and Marine “Candidatus Manganitrophaceae” Species Implies Their Worldwide Reach in Manganese Chemolithoautotrophy
Source: mBio. 2022 Mar 14;13(2):e03421-21. doi: 10.1128/mbio.03421-21 (PMC9040806; doi:10.1128/mbio.03421-21)
Supplement: TABLE S5 [file mbio.03421-21-st005.docx]

**Supplementary Table 5. Genome statistics of the publicly available metagenome-assembled genomes used in this study.** The MAG IDs for the family *Candidatus* Manganitrophaceae were highlighted in pink, with the text of the marine genus in brown; other members in the order *Candidatus* Troglogloeales are highlighted in blue.

| **MAG ID** *(NCBI Assembly or MAG ID from genomic catalog of Earth’s microbiomes)* | **Completeness** | **Contamination** | **Strain heterogeneity** |
| --- | --- | --- | --- |
| **GCA_013151935** | 91.31 | 2.23 | 0 |
| **GCA_012960925** | 96.76 | 1.82 | 50 |
| **GCA_015659975** | 96.76 | 0.91 | 0 |
| **GCA_016200325** | 97.73 | 3.64 | 0 |
| **GCA_004297235** | 93.35 | 3.64 | 20 |
| **GCA_005239595** | 91.55 | 7.83 | 0 |
| **GCA_005239745** | 95.22 | 0 | 0 |
| **GCA_005239925** | 82.94 | 1.89 | 33.33 |
| **GCA_016201925** | 73.59 | 0 | 0 |
| **GCA_016195485** | 86.51 | 0 | 0 |
| **GCA_016194305** | 95.85 | 2.73 | 0 |
| **GCA_016178565** | 81.53 | 3.69 | 0 |
| **GCA_016195515** | 94.94 | 1.82 | 0 |
| **GCA_016178585** | 97.67 | 2.05 | 33.33 |
| **GCA_016201875** | 81.31 | 1.21 | 33.33 |
| **GCA_016178525** | 66.48 | 4.77 | 0 |
| **GCA_011331265** | 79.84 | 4.6 | 0 |
| **GCA_016201765** | 84.55 | 1.87 | 0 |
| **GCA_001803875** | 62.65 | 0.91 | 0 |
| **GCA_016201825** | 89.29 | 3.69 | 16.67 |
| **GCA_016234185** | 80.4 | 3.64 | 25 |
| **GCA_016194285** | 89.94 | 0 | 0 |
| **GCA_016201865** | 93.12 | 0.91 | 0 |
| **GCA_016198945** | 67.32 | 2.11 | 40 |
| **GCA_016200255** | 93.07 | 3.79 | 33.33 |
| **GCA_016212145** | 57.52 | 0 | 0 |
| **GCA_016208775** | 59.77 | 5.43 | 44.44 |
| **GCA_001805165** | 61.74 | 1.82 | 0 |
| **GCA_016234155** | 65.45 | 0 | 0 |
| **GCA_016212215** | 95 | 3.18 | 0 |
| **GCA_016217665** | 77.1 | 1.82 | 50 |
| **GCA_016215185** | 71.68 | 3.29 | 83.33 |
| **GCA_016214375** | 68.07 | 0.91 | 100 |
| **GCA_003477265** | 86.36 | 6.36 | 12.5 |
| **GCA_001805025** | 71.23 | 0.91 | 0 |
| **GCA_001805205** | 80.55 | 1.82 | 0 |
| **GCA_003508715** | 74.29 | 3.91 | 50 |
| **GCA_001803925** | 64.39 | 0.91 | 0 |
| **GCA_001805235** | 65.18 | 0.91 | 0 |
| **GCA_016195585** | 81.91 | 0.97 | 0 |
| **GCA_016212265** | 53.58 | 0.91 | 0 |
| **GCA_016212285** | 62.29 | 3.94 | 28.57 |
| **GCA_001803815** | 60.95 | 0.91 | 0 |
| **GCA_016214425** | 72.06 | 3.18 | 60 |
| **GCA_003454665** | 78.46 | 1.04 | 75 |
| **GCA_001805055** | 93.18 | 1.42 | 0 |
| **GCA_001805045** | 56.97 | 0.91 | 0 |
| **GCA_001803725** | 70.61 | 0.06 | 0 |
| **GCA_003483085** | 82.92 | 3.24 | 16.67 |
| **GCA_003475985** | 58.94 | 0.51 | 0 |
| **GCA_001803845** | 53.41 | 0 | 0 |
| **GCA_013334545** | 93.33 | 3.96 | 33.33 |
| **GCA_013336065** | 95.4 | 5.74 | 22.22 |
| **3300027902_59** | 67.02 | 0.91 | 0 |
| **3300028028_11** | 95 | 3.18 | 25 |
| **3300027905_39** | 96.82 | 2.73 | 0 |
| **GCA_001803705** | 67.05 | 0.91 | 0 |
| **GCA_001805085** | 80.46 | 2.73 | 0 |
| **GCA_001803765** | 90.81 | 6.72 | 0 |
| **3300017935_14** | 82.56 | 0.91 | 0 |
| **GCA_016212105** | 97.27 | 0.91 | 0 |
| **GCA_016222885** | 98.18 | 0.96 | 0 |
| **GCA_016217625** | 73.05 | 1.89 | 66.67 |
| **GCA_001803795** | 89.55 | 0.91 | 0 |
| **GCA_016178245** | 66.76 | 0 | 0 |
| **GCA_016195465** | 92.05 | 1.36 | 100 |
